# DKG-Zertifizierung kinderonkologischer Zentren – ein weites Feld …

**DOI:** 10.1007/s00103-020-03112-z

**Published:** 2020-03-11

**Authors:** Melchior Lauten, Udo Kontny, Michaela Nathrath, Martin Schrappe

**Affiliations:** 1 Klinik für Kinder- und Jugendmedizin, Bereich Kinderhämatologie und Onkologie, Universitätsklinikum Schleswig-Holstein, Campus Lübeck, Ratzeburger Allee 160, 23538 Lübeck, Deutschland; 2 Klinik für Kinder- und Jugendmedizin, Sektion Pädiatrische Hämatologie, Onkologie und Stammzelltransplantation, Uniklinik RWTH Aachen, Aachen, Deutschland; 3 Pädiatrische Hämatologie und Onkologie, Psychosomatik und Systemerkrankungen, Klinikum Kassel, Kassel, Deutschland; 4 Klinik für Kinder- und Jugendmedizin I, Universitätsklinikum Schleswig-Holstein, Campus Kiel, Kiel, Deutschland

**Keywords:** Kinderonkologie, Zentrum, DKG, GPOH, Zertifizierung, Fallzahl, Childhood oncology, Paediatric oncology centre, DKG, GPOH, Certification, Case number

## Abstract

Die Gesellschaft für Pädiatrische Onkologie und Hämatologie (GPOH) und die Deutsche Krebsgesellschaft (DKG) haben Kriterien für die DKG-Zertifizierung der Kinderonkologie erarbeitet, nach denen 2017 in Deutschland erstmals pädiatrisch-onkologische Abteilungen zertifiziert wurden. Das Ziel der DKG-Zertifizierung ist „die Vereinheitlichung und die transparente Darstellung der Versorgungsqualität für pädiatrische Patientinnen und Patienten mit hämato-onkologischen Erkrankungen“, wie 2018 in einer Veröffentlichung von Mensah et al. dargestellt. Die Auswahl der Zertifizierungskriterien hat innerhalb der GPOH zu einer intensiven Diskussion darüber geführt, inwieweit die Kriterien für sich genommen einer wissenschaftlichen Überprüfbarkeit standhalten und damit valide Parameter für die Bestimmung der Versorgungsqualität in der Kinderonkologie in Deutschland darstellen.

Wir haben untersucht, ob aus der Arbeit von Mensah et al. valide Folgerungen für das deutsche Gesundheitssystem ableitbar sind. Dabei zeigt sich, dass die momentan definierten DKG-Zertifizierungskriterien für die Kinderonkologie in Deutschland in kritischen Bereichen einer fundierten wissenschaftlichen Grundlage entbehren.

Diese Arbeit stellt Fallzahlen als Maß für Qualität in der Kinderonkologie infrage und regt die Entwicklung alternativer Kriterien für die Überprüfbarkeit von Qualität in der deutschen Kinderonkologie an.

## Abstract (en)

The German Society of Paediatric Oncology and Haematology (GPOH) and the German Cancer Society (DKG) have defined criteria for DKG certification of paediatric oncology departments. Since 2017, several paediatric oncology departments have already been certified according to these criteria. DKG certification aims for the harmonized and transparent presentation of the quality of care of paediatric oncology patients, as described by Mensah et al. The definition of certification criteria led to controversies within the GPOH about how far the criteria themselves would withstand scientific verifiability.

We critically reviewed the paper by Mensah et al. asking whether valid conclusions for the German health system could be drawn from it. We found that currently defined criteria for DKG certification of paediatric oncology departments lack scientific evidence for German paediatric cancer centres in critical aspects.

This article challenges case numbers as a parameter for the measurement of quality of care in German paediatric oncology. We try to contribute to an open discussion about alternative criteria for ensuring quality of care in German paediatric oncology departments.

## Einleitung

Die Zertifizierung onkologischer (Organ‑)Zentren hat seit ihrer Einführung 2011 zu einer verbesserten Sichtbarkeit onkologischer Expertise in Deutschland beigetragen. Im November 2018 wurde eine Stellungnahme der Deutschen Krebsgesellschaft e. V. (DKG) und der Gesellschaft für Pädiatrische Onkologie und Hämatologie (GPOH) zur Einführung der DKG-Zertifizierung kinderonkologischer Zentren in Deutschland veröffentlicht [1]. In dieser Stellungnahme wird beschrieben, dass die Zertifizierung kinderonkologischer Zentren eine logische Weiterentwicklung der historisch gewachsenen und funktionierenden Zusammenarbeit kinderonkologischer Zentren in Deutschland ist und dass das Zertifizierungsverfahren als solches für die Kinderonkologie einen Mehrwert zu bestehenden Rahmenbedingungen, vorgegeben durch die Richtlinien des Gemeinsamen Bundesausschusses (G-BA), bietet. Diese Feststellung hat seither zu einer lebhaften Diskussion geführt. Daher soll mit diesem Beitrag die aktuelle Datenlage bewertet und in den Kontext des Deutschen Gesundheitssystems gestellt werden.

Die Stellungnahme beginnt mit einem historischen Abriss der Entwicklung kinderonkologischer Strukturen und beschreibt, dass die Entwicklung der DAL (Deutsche Arbeitsgemeinschaft für Leukämie-Forschung und -Behandlung im Kindesalter e. V.), der GPO (Gesellschaft für Pädiatrische Onkologie) und schließlich der GPOH dem Ziel folgte, qualitätssichernde Maßnahmen für die Kinderonkologie und später auch die Kinderhämatologie zu implementieren und zu evaluieren. Im Mai 2006 mündete diese Entwicklung in einer Richtlinie des G‑BA, die erstmals Infrastrukturmerkmale für kinderonkologische Zentren definierte und diese ab Januar 2007 verpflichtend einführte [2]. Im Abschlussbericht des G‑BA wurden geeignete Qualitätsindikatoren und geprüfte Prozessabläufe zur Überprüfung der Vereinheitlichung von Versorgungsqualität für die Kinderonkologie gefordert, schließlich jedoch nicht in die entsprechende Richtlinie mit aufgenommen. Daher seien diese, so die Autoren der Stellungnahme, in der Folge in enger Kooperation zwischen DKG und GPOH auf der Grundlage der G‑BA-Richtlinie entwickelt und schließlich in einem Zertifizierungsmodul zusammengefasst worden. Das Zertifizierungsmodul verstehe sich dabei nicht als Umsetzungsinstrument der G‑BA-Richtlinie, sondern wolle in seinen Anforderungen darüber hinausgehen.

Die Notwendigkeit für die Einführung von Qualitätsindikatoren zur Überprüfung von Versorgungsqualität leiten die Autoren im Wesentlichen aus einem Reviewartikel von Knops et al. ab, in dem Knops et al. zu der Schlussfolgerung kommen: „This systematic review provides support for the statement that higher volume hospitals, higher case volume providers, and specialised hospitals are related to the better outcome in paediatric oncology. No studies reported a negative effect of a higher volume“ [3]. Zudem wird eine Richtlinie des National Health Service (NHS) zitiert, in der „der NHS … die Verbesserung der Überlebensraten pädiatrisch-onkologischer Patienten in England und Wales in den letzten 30 Jahren unter anderem auf eine zentralisierte Betreuung dieser Kinder in spezialisierten Zentren zurück[führe]“ [4].

Es stellt sich also die Frage, ob Untersuchungen und Richtlinien aus dem britischen Gesundheitssystem eine ausreichende wissenschaftliche Grundlage für Schlussfolgerungen in Bezug auf die spezifische Situation der Kinderonkologie in Deutschland darstellen.

## Die Richtlinie des NHS zur Verbesserung des Überlebens von Kindern und Jugendlichen mit Krebserkrankung

Die Richtlinie „Improving Outcomes in Children and Young People with Cancer“ wurde 2005 veröffentlicht und definierte erstmals Diagnose‑, Therapie- und Versorgungspfade für Kinder und Jugendliche, die in England und Wales mit Krebs erkrankten [4]. Als Leitlinienprinzipien definierten die Autoren u. a., dass alle Leitlinienempfehlungen einer nachvollziehbaren Evidenz folgen sollten und in der Versorgung der Patienten ein nachhaltiges Gleichgewicht zwischen Zentralisation und Dezentralisation herrschen sollte [4, S. 9–10]. In England und Wales waren die Überlebensraten der erkrankten Kinder und Jugendlichen zu jener Zeit im Vergleich zu Deutschland deutlich schlechter [4]. Zudem wurden im Bereich des NHS verhältnismäßig weniger Kinder und Jugendliche in kontrollierten klinischen Studien behandelt als in anderen europäischen Ländern [5, 6]. Daher erhofften sich die Autoren der NHS-Richtlinie, das Überleben der Patienten in England und Wales u. a. durch eine höhere Einschlussrate in Therapiestudien zu verbessern [4, S. 18–19]. Die Schaffung von Netzwerken („cancer networks“) sollte dabei gewährleisten, dass die Behandlung der Kinder in spezialisierten Kliniken mit ausreichend Behandlungszeit durch Spezialisten („adequate specialist time“) erfolgen könne [4, S. 28–29]. Die wurde auch in Bezug auf die chirurgische Expertise gefordert: „Diagnostic biopsy or definitive surgery … should be carried out by surgeons appropriately trained … working in a center with appropriate support …“ [4, S. 42]. „Appropriate training“ und „appropriate support“ wurden dabei nicht weiter definiert.

## Analyse der gemeinsamen Stellungnahme der DKG und der GPOH

In der Stellungnahme der DKG und der GPOH aus dem Jahr 2018 wird festgehalten, dass „eine adäquate Expertise für die Behandlung [von Kindern und Jugendlichen mit Krebs] nur durch eine Zentralisierung der Versorgung an ausgewiesenen Zentren zu erreichen [sei]“. Die Autoren berufen sich dabei wesentlich auf den oben genannten Reviewartikel von Knops et al. aus dem Jahr 2013. In diesen Reviewartikel wurden Studien aufgenommen, wenn mindestens 90 % der Studienpatienten einem pädiatrischen Kollektiv entsprachen. Nach Sichtung von 731 Studien wurden am Ende 14 Studien eingeschlossen und ausgewertet. Durch die Autoren wurde eine Kategorisierung nach Zentrumsgröße („high volume“ und „low volume“) vorgenommen und als Trennwert für „high“ bzw. „low volume“ eine Fallzahl von *n* = 5 Patienten pro Jahr und Zentrum (oder Behandler) festgelegt [3]. Eine Patientenanzahl von mehr als fünf Patienten pro Zentrum bzw. Behandler definierte dabei „high volume“.

Alle eingeschlossenen Studien dieses Reviewartikels haben ein retrospektives Design, sieben Studien kommen aus Großbritannien (sechs davon aus derselben Arbeitsgruppe; [7–13]), fünf Studien aus den USA [14–18] und eine (monozentrische) Studie aus Kanada [19]. Eine weitere Studie, die Ergebnisse nach allogener Stammzelltransplantation (SZT) bei akuter myeloischer Leukämie (AML), akuter lymphoblastischer Leukämie (ALL) und chronischer myeloischer Leukämie (CML) in den Jahren 1983–1987 in Abhängigkeit von der Zentrumsgröße analysiert, zeigt Daten einer internationalen Kooperation ohne deutsche Beteiligung [20]. Sechs der 14 Studien untersuchen Effekte bei hämatologischen Neoplasien ohne [8–11, 14] bzw. mit allogener SZT [20], drei bei soliden Tumoren außerhalb des zentralen Nervensystems (ZNS; [12, 16, 17]), drei bei soliden Tumoren des ZNS [15, 18, 19] und zwei bei einem gemischten Kollektiv, das Patienten mit hämatologischen oder soliden Neoplasien aus den Jahren 1968–1984 einschließt [7, 13]. Sechs der 14 Studien zeigen Daten aus multivariaten Analysen [12, 15–17, 19, 20] bei Patienten mit soliden Tumoren außerhalb des ZNS (*n* = 3), soliden ZNS-Tumoren (*n* = 2) bzw. HLA-identer Geschwister-SZT (*n* = 1).

In den Studien wurden Hinweise gefunden, dass Patienten mit ALL zwischen 1968–1982 im Großraum Newcastle in „central hospitals“ erfolgreicher behandelt wurden als in „peripheral hospitals“ [13]. Dies galt in derselben Studie nicht für Patienten mit AML, Non-Hodgkin-Lymphom (NHL), Hodgkin-Lymphom, Osteosarkom oder ZNS-Tumoren. Im Zeitraum 1971–1982 zeigte sich zudem ein besseres Überleben für ALL-Patienten in Behandlungszentren mit „großem“ Patientenaufkommen („high volume hospitals“; >6 ALL-Patienten/Jahr), wobei dies für eine Zeit untersucht wurde, in der das 5‑Jahres-Überleben in den größeren Zentren zwischen 46 % (1971–1973) und 67 % (1980–1982) lag [9]. Der gefundene Unterschied galt dabei nicht für Patienten, die in Studien behandelt wurden. Zudem zeigte sich, dass der Effekt ab 1977 nur noch im Vergleich zu Zentren nachweisbar war, in denen <1 ALL-Patient/Jahr behandelt wurde [9]. Ähnliches wurde für Patienten mit AML und NHL gefunden: Es gab Hinweise darauf, dass das Überleben in pädiatrisch-onkologischen Krankenhäusern besser war als in anderen Krankenhäusern [7, 10]. Zudem zeigte sich eine erhöhte frühe Todesrate für AML-Patienten, die nicht in Studien eingeschlossen wurden. Die Autoren führten dies darauf zurück, dass vermutlich schwer kranke Patienten a priori weniger in Studien einschlossen wurden [8].

Bei Patienten mit Neuroblastom oder Wilmstumor war in Bezug auf die Anzahl der Operationen pro Operateur (>/<5) kein Unterschied im 5‑Jahres- und 10-Jahres-Überleben erkennbar. Es zeigte sich aber, dass Kinder, die in Krankenhäusern der Childhood Oncology Group (COG) behandelt wurden, weniger häufig an ihrer Erkrankung verstarben [16].

Bei Patienten mit Osteosarkom oder Ewingsarkom ist die Datenlage der ausgewerteten Studien uneinheitlich. In einer englischen Studie, in der die Behandlungserfolge (3-Jahres-Überleben) in „Kinderonkologischen Zentren“ („paediatric oncology centres“ [POC]), in „anderen Lehrkrankenhäusern“ („other teaching centres“ [TC]) und in „anderen Nicht-Lehrkrankenhäusern“ („other non-teaching centres“ [NTC]) verglichen wurden, zeigte sich auf der einen Seite, dass Kinder mit Osteosarkom <15 Lebensjahren im Zeitraum 1977–1984 in POC gleich gut behandelt wurden wie in TC und in NTC (Univariatanalyse; [7]). Auf der anderen Seite zeigte dieselbe Studie, dass Kinder mit Ewingsarkom in POC und in NTC besser behandelt wurden als in TC. Eine zweite Studie derselben englischen Arbeitsgruppe untersuchte, ob es auch im Zeitraum 1980–1994 für Osteosarkompatienten und Ewingsarkompatienten einen Zentrumseffekt in Bezug auf das 5‑Jahres-Überleben gab. Die Zentren wurden kategorisiert in supraregionale Bone Tumour Services (BTS), in Krankenhäuser der United Kingdom Children’s Cancer Study Group (UKCCSG), in TC und in NTC. Die Schlussfolgerung nach Multivariatanalyse bleibt vage: „In the present study … there was some indication that survival was higher at specialist centres than at other hospitals, but the differences were not always statistically significant“ [12]. Eine Überlebensanalyse nach Fallzahlen wurde in keiner der beiden Studien vorgenommen.

Für Kinder, die im Alter <2 Jahren an einem Hirntumor erkrankten, wurden starke Hinweise gefunden, dass neurochirurgische Erfahrung sowohl Einfluss auf die Rate an Resektionen ohne Residulatumor (R0-Resektionen) als auch Einfluss auf das Gesamtüberleben der Kinder haben kann [15, 19]. Zudem zeigte sich, dass die Resektionsrate bei Neurochirurgen, die 4,9 Patienten/Jahr operieren, im Vergleich zu Kollegen, die 1,9 Patienten/Jahr operieren, vermutlich besser ist [18].

Insgesamt zeichnen die 14 Studien, die ganz überwiegend Daten aus der Zeit bis 2006 analysieren, ein heterogenes Bild heterogener Outcomeparameter in unterschiedlichen Gesundheitssystemen aus einer Zeit, in der die Heilungschancen für Kinder mit onkologischen Erkrankungen noch deutlich unvorteilhafter waren als heute.

## Beeinflussen Zentrumsgröße, Fallzahlen und das Ausmaß der Spezialisierung einer Klinik die Prognose von Kindern und Jugendlichen mit Krebs?

Eine kürzlich veröffentlichte Studie untersuchte, in welchem Maße Zentralisierung von Behandlungszentren zum Therapieerfolg bei Kindern mit Krebserkrankung beiträgt [21]. Es wurden 4415 Kinder eingeschlossen, die zwischen 2000–2007 in Belgien, Bulgarien, Finnland, Irland, den Niederlanden und Slowenien erkrankt waren. Die Autoren differenzierten große von kleinen Behandlungszentren durch zwei Definitionen: Alle Zentren eines Landes wurden nach Anzahl der jährlich behandelten Patienten derselben Tumorentität nebeneinander aufgetragen. Immer dort, wo sich die Patientenanzahl von zwei nebeneinander aufgetragenen Zentren um mindestens 100 % unterschied, lag die Trennlinie zwischen großen und kleinen Zentren. Sofern es diesen Trennpunkt nicht gab, wurde die Gruppe von Zentren als große Zentren bezeichnet, die mindestens die Hälfte der Patienten eines Landes behandelt hatten. Mit dieser Definition wurden pro Land 1–5 große Zentren definiert, ein erheblicher Anteil der Zentren hatte <10 Patienten/Jahr behandelt. Die Autoren zeigten in einer gepoolten Analyse einen signifikanten Überlebensvorteil von Patienten, die wegen hämatologischer Neoplasien oder ZNS-Tumoren in größeren Zentren behandelt wurden. Sobald diese Analyse jedoch ungepoolt, d. h. für jedes Land getrennt durchgeführt wurde, verloren die Ergebnisse ihre Signifikanz [21].

Zusätzlich zu der oben beschriebenen Analyse wurde untersucht, ob Unterschiede im Überleben nachweisbar waren, wenn eine Fallzahl von *n* = 30 als Trenngröße zwischen großen und kleinen Behandlungszentren angenommen wurde. In dieser Analyse war der bessere Behandlungserfolg bei hämatologischen Neoplasien nicht mehr nachweisbar. Die Trenngröße von *n* = 30 wurde gewählt, da die Autoren diese Anzahl als europäischen Standard ansahen. Sie berufen sich dabei auf eine Veröffentlichung von Kowalczyk et al. [22], in der jedoch weder über Fallzahlen insgesamt noch über eine trennende Fallzahl von 30 gesprochen wird. Aus den insgesamt heterogenen Daten folgern die Autoren, dass Fallzahlen als alleinige Ursache einer regional unterschiedlichen Versorgungsqualität nicht gelten können.

Auch in Deutschland haben sich Fallzahlen als Werkzeug für die Beurteilung von Versorgungsqualität in der Kinderonkologie bislang nicht überzeugend nachweisen lassen. In der Studie SIOP 93-01 (International Society of Paediatric Oncology) zur Behandlung von Nephroblastomen zeigte sich zwar, dass die postoperative Behandlungsintensität bei Patienten mit unilateralem Nephroblastom vom Erfahrungsgrad des Chirurgen abhängig ist. Es zeigte sich jedoch nicht, dass die Anzahl der pro Jahr und Zentrum operierten Patienten einen Einfluss auf das 5‑Jahres- und 10-Jahres-Überleben hat [23]. Da Daten zu therapieassoziierten Langzeitfolgen für diese Studie fehlten, konnten die Autoren nicht zeigen, ob in der intensiver behandelten Gruppe langfristig mehr Therapietoxizität auftrat. Dies wäre biologisch jedoch plausibel.

Zahlen, die eine Verbesserung der Versorgungsqualität in Abhängigkeit von der Zentrumsgröße bei ZNS-Tumoren in Deutschland belegen, fehlen. In der inzwischen abgeschlossenen Studie HIT 2000 zur Behandlung von Medulloblastomen, Ependymomen und primitiven neuroektodermalen Tumoren (PNET) im Kindesalter wurde zwar prospektiv untersucht, ob die Größe eines Behandlungszentrums (>/<4 HIT 2000 Studienteilnehmer/Jahr) einen Einfluss auf das Überleben und das progressionsfreie Überleben hat. Die Ergebnisse dieser Subanalyse liegen jedoch noch nicht vor. Sie werden im Bereich des deutschen Gesundheitswesens erstmals zeigen, ob eine Diskussion über operative Fallzahlen bei neuroonkologischen Erkrankungen notwendig ist, um die Patientenversorgung weiter zu verbessern. Da die HIT-2000-Studie über einen langen Zeitraum lief, kann sie möglicherweise sogar im Hinblick auf das Inkrafttreten des G‑BA-Beschlusses bzw. seine Auswirkung auf die Versorgungsqualität hin ausgewertet werden und damit Hinweise geben, welchen Einfluss Standardisierungen in einem gut funktionierenden System grundsätzlich haben können.

## Einordnung in den Kontext der Kinderonkologie in Deutschland

Systeme, die Prozesse und Behandlungsstränge vereinheitlichen, können zur Verbesserung der Versorgungsqualität in der Patientenversorgung führen [24]. Dies war die Grundidee, die in Deutschland in den 1970er-Jahren zur Gründung der DAL führte und dazu beitrug, dass zentrumsübergreifende Kommunikationswege etabliert und Behandlungsstrategien definiert wurden. Neben anderen war es auch diese Initiative, die den Grundstein legte für die Etablierung moderner Therapiestudien, welche seither weit über Deutschland und Europa hinaus Anerkennung und Anwendung finden.

Seitdem der G‑BA seine Evaluation der pädiatrischen Onkologie vorgelegt und Kriterien für die Versorgungsqualität in der pädiatrischen Onkologie festgelegt hat, haben sich die Versorgungsstrukturen in Deutschland weiter vereinheitlicht. Dabei ist die Anzahl der kinderonkologischen Behandlungszentren in zeitlichem Zusammenhang gesunken: Während im Zeitraum 1999–2008 noch Kinder aus 83 Zentren an das Deutsche Kinderkrebsregister (DKKR) gemeldet wurden [25], erhielt das DKKR im Jahr 2017 Meldungen nur noch aus 63 Zentren [26]. Die Abnahme der Anzahl der Behandlungszentren war mutmaßlich Folge der Nichterfüllbarkeit der G‑BA-Strukturvorgaben.

Ob damit eine Verbesserung der Versorgungsqualität einherging, ist bislang nicht nachgewiesen. Daher ist das Ziel des Zertifizierungsprozesses der DKG, Versorgungsqualität überprüfbar zu machen, von hoher Wichtigkeit. Durch wissenschaftliche Evidenz abgesicherte Qualitätsindikatoren, die dafür zur Hilfe genommen werden können, existieren, wie oben dargestellt, für die Kinderonkologie in Deutschland nicht. Die sehr gute Beurteilung, die der G‑BA der Kinderonkologie in seinem Gutachten ausstellte, beruhte auf der Analyse aller kinderonkologischen Zentren in der Zeit vor 2006, unabhängig von deren Größe. Kleinere Zentren, definiert als solche Zentren, die <30 Patienten im Jahr versorgen und daher in Zukunft vom Zertifizierungsprozess der DKG ausgeschlossen sein sollen, trugen zu dieser Einschätzung zu etwa 25 % und mit etwa 500 Kindern bei [26].

Führt der DKG-Zertifizierungsprozess zukünftig zur Lenkung von Patientenströmen in zertifizierte Zentren nach derzeitigen DKG-Kriterien, wie dies durch eine Infobox am Ende des Artikels von Mensah et al. suggeriert wird, wird die Versorgung dieser 500 Kinder/Jahr von den zertifizierten Zentren mit übernommen werden und werden die Familien dieser Kinder eine heimatfernere Behandlung als bisher in Anspruch nehmen müssen. Ob im selben Maße das Behandlungsteam weitere Wege auf sich nehmen würde, um die medizinische Versorgung der zusätzlichen Patienten in den aufnehmenden Zentren zu gewährleisten, und ob die jeweiligen aufnehmenden Behandlungszentren ihre Bettenkapazität in entsprechendem Maße aufstocken könnten, wurde bislang nicht öffentlich diskutiert.

Es erscheint grundsätzlich nicht plausibel, Fallzahlen als Surrogatparameter für Qualität zu definieren, sofern diese Parameter nicht überprüft sind. Knops et al. forderten daher: „The threshold that indicates the number of childhood cancer patients needed to treat to benefit from the volume effect remains unclear. More studies including a greater number of children with the same tumour type have to be carried out …“ [3]. Im Bereich der GPOH-Kliniken gilt dies in besonderer Weise, da es hier bereits zum jetzigen Zeitpunkt Standard ist, Patienten annähernd ausnahmslos in großen klinischen Studien zu behandeln und lokale Therapieentscheidungen durch externe Referenzgremien überprüfen zu lassen. Patienten, die seltene Tumoroperationen oder Strahlentherapien benötigen, werden für diese Interventionen regelmäßig in entsprechende Referenzzentren verlegt. Dass diese Patienten, die aus qualitativen Gründen außerhalb des ursprünglich behandelnden Zentrums operiert werden, nun nach Maßgabe der Zertifizierungskriterien in die Fallzahlberechnung des verlegenden Zentrums eingerechnet werden dürfen, um dort chirurgische oder strahlentherapeutische Expertise nachzuweisen, ist zwar ein freundliches Entgegenkommen gegenüber den kleineren Zentren, kehrt die inhaltliche Argumentation insgesamt allerdings ad absurdum.

Qualitätsstandards, wie sie im Bereich des NHS gefordert werden, sind im Bereich des deutschen Gesundheitssystems bereits zum jetzigen Zeitpunkt weitestgehend erfüllt, daher müssten Qualitätsindikatoren zur Überprüfung der hier vorhandenen hohen Versorgungsqualität in einem ersten Schritt auf dem Boden der gegebenen Strukturen und unter Führung der GPOH z. B. durch ein Delphi-Verfahren definiert und nach Möglichkeit prospektiv geprüft werden. Dies sahen die Autoren der gemeinsamen Stellungnahme von DKG und GPOH ebenfalls und forderten: „Es ist daher essenziell zu prüfen, ob und in welchem Maße die qualitative Versorgung auch tatsächlich bei den Patienten ankommt. Erst hier zeigt sich die tatsächliche Güte der vorab definierten Prozesse“ [1].

Patienten, die innerhalb von klinischen Studien behandelt werden, können gegenüber Patienten, die nicht in Studien behandelt werden, einen Vorteil in Bezug auf den Behandlungserfolg zeigen [27]. Sobald jedoch nicht die überwiegende Mehrzahl der Patienten in klinische Studien rekrutiert wird, zeigt sich dieser Effekt nicht mehr [28]. Aus unserer Sicht könnten daher z. B. die Konsequenz und die zeitliche Nähe, mit denen Patienten mit allen erforderlichen Daten initial und im Verlauf durch die Studienkliniken an die Studienleitungen gemeldet werden, als Maß für Qualität in der Versorgung prospektiv evaluiert werden. Auch die Erfassung und der Umgang mit Therapieabweichungen, die bei Patienten außerhalb von Studien auftreten, könnten hier Hinweise auf die Qualität interner Versorgungsstrukturen geben.

In der aktuellen Leitlinie der Arbeitsgemeinschaft der Wissenschaftlichen Medizinischen Fachgesellschaften e. V. (AWMF) für die Diagnostik und Therapie bei Kindern mit onkologischer Grunderkrankung, Fieber und Granulozytopenie (mit febriler Neutropenie) außerhalb der allogenen Stammzelltransplantation [29] wird gefordert, dass „Kinderonkologische Behandlungszentren … personell und strukturell-organisatorisch so aufgestellt und ausgestattet sein [sollen], dass sie jederzeit alle … erforderlichen medizinischen Maßnahmen der Diagnostik und Therapie zeitgerecht anbieten können“. Diese Forderung nimmt den Grundsatz der Richtlinie des NHS auf, die ein „nachhaltiges Gleichgewicht zwischen Zentralisation und Dezentralisation“ für die Verbesserung der Versorgung pädiatrisch-onkologischer Patienten definiert. Die Zeit bis zur ersten Gabe eines Antibiotikums („time to antibiotics“ [TTA]) ist bei Kindern mit febriler Neutropenie ein Marker dafür, wie oft im Verlauf der Fieberepisode eine intensivmedizinische Behandlung benötigt wird [30]. Dabei misst die TTA, wie gut interne Klinikabläufe strukturiert sind und wie vertraut der jeweils behandelnde Arzt mit der Einschätzung kritischer Situationen bei kinderonkologischen Patienten ist. Sie wäre daher aus unserer Sicht ebenfalls ein Marker, um Versorgungsqualität in kinderonkologischen Zentren zu messen.

Die bislang benannten Marker zielen auf den Endpunkt Überleben. In Zeiten, in denen das bloße Überleben jedoch nicht mehr für jede Erkrankungssubgruppe ein valider Endpunkt ist, geraten alternative Endpunkte in den Fokus. Solche Endpunkte können beispielsweise die Lebensqualität nach Abschluss einer onkologischen Behandlung sein oder auch die Fähigkeit, schwierige Lebenssituationen ohne anhaltende psychische Beeinträchtigung zu überstehen (Resilienz). Strukturen, die darauf abzielen, Lebensqualität zu fördern und Resilienz zu stärken, sind vor allem im psychoonkologischen Dienst angesiedelt und werden vom G‑BA für kinderonkologische Zentren gefordert. Darüber hinausgehende Unterstützungen z. B. im schulischen Bereich, auch unter Nutzung moderner Medien, können vermutlich dazu beitragen, die soziale Teilhabe onkologisch erkrankter Kinder zu erhöhen. Ob solche Maßnahmen zur Verbesserung der posttherapeutischen Lebensqualität insgesamt beitragen, sollte prospektiv geprüft werden.

Eine Fallzahldefinition, wie sie momentan durch die DKG gefordert wird, wird unzweifelhaft zur Reduktion kinderonkologischer Zentren in Deutschland führen. Auch universitäre Zentren könnten davon betroffen sein. Damit ginge langfristig nicht nur die vorhandene Expertise in der klinischen Routine und in der pädiatrischen und operativen Notfallversorgung verloren, die Zentralisierung würde sich auch unmittelbar auf die Ausbildung junger Kollegen und auf Forschung und Lehre auswirken und damit einen Prozess initiieren, der international den Standort Deutschland nicht stärken, sondern schwächen würde.

Unabhängig von der Diskussion um Fallzahlen, ergibt sich jedoch bei der Zertifizierung kinderonkologischer Zentren nach momentaner Maßgabe der DKG ein zusätzlich kritischer Aspekt, da kinderonkologische Abteilungen für sich genommen die Zertifizierungskriterien zwar erfüllen, aber dennoch vom Zertifizierungsprozess ausgeschlossen sein könnten. Grund dafür ist die Tatsache, dass die Zertifizierung der Kinderonkologie als Modul vorgenommen wird und damit abhängig von der Existenz onkologischer Zentren innerhalb der eigenen Institution ist. Insbesondere in bevölkerungsschwachen Flächenländern, in denen onkologische Zentren allein aufgrund der Bevölkerungsdichte schwierig zu implementieren sind, droht damit eine regionale Unterversorgung.

Im Jahr 2018 ist in den Niederlanden die Versorgung kinderonkologischer Patienten radikal zentralisiert und im Princess Máxima Center in Utrecht zusammengeführt worden. Damit sind die Niederlande momentan das einzige größere europäische Land, in dem komplexe Krankenversorgung, klinische und experimentelle Spitzenforschung sowie die Ausbildungen junger Kinderonkologen an einem Zentrum vereint sind. Unzweifelhaft verbindet sich mit dieser Zentralisierung die Hoffnung auf Qualitätsgewinn durch die enge Verzahnung von Patientenversorgung, Forschung und Lehre. Es wird daher spannend sein zu beobachten, ob sich diese Hoffnung langfristig in einer Steigerung der Versorgungsqualität, der wissenschaftlichen Expertise und nicht zuletzt der Patientenzufriedenheit wird messen lassen können.

Das G‑BA-Gutachten endete an dem Punkt, an welchem weitere validierte Forderungen ohne die Durchführung zusätzlicher Studien nicht mehr geäußert werden konnten. Wir nehmen dies auf und regen an, eine Fallzahlnennung für die DKG-Zertifizierung der Kinderonkologie so lange auszusetzen, bis fundierte Daten verfügbar sind, die die Versorgungsqualität in Deutschland überprüfbar machen. Entsprechende Studien könnten unter Federführung der GPOH untersuchen, wie in den momentan G‑BA-konform betriebenen kinderonkologischen Zentren die Zeit bis zur ersten Antibiotikagabe („time to treat“) bei febriler Infektion, die Studienadhärenz, die Dokumentation der Behandlung oder auch die Teilnahme von Vertretern der operativen Fächer an Fortbildungen/Studientreffen ist.

## Ethische Befangenheiten

Einige der Autoren dieses Papers stehen im Interessenkonflikt zwischen dem Versuch, die Qualität der kinderonkologischen Versorgung in Deutschland zu verbessern, gleichzeitig jedoch die momentan geforderten Fallzahlen für die DKG-Zertifizierung im Bereich der eigenen Institution nicht sicher in jedem Jahr zu erreichen.

Die Autoren der Veröffentlichung von Mensah et al. standen ungewollt im Interessenkonflikt zwischen ihrer beruflichen Tätigkeit (Angestellte der DKG bzw. Vertreter großer Kinderonkologien in Deutschland) und der gemeinsamen Tätigkeit der Vorbereitung und Implementierung des DKG-Zertifizierungsmoduls. Eine erfolgreiche Implementierung des Zertifizierungsmoduls würde unweigerlich mit finanziellen Vorteilen der DKG (kostenpflichtiger Zertifizierungs- und Rezertifizierungsprozess) und möglicherweise auch der Universitätsklinika einhergehen (Zunahme der Patientenströme).

Ein weiterer Interessenkonflikt wird aus der Zusammensetzung der DKG-Zertifizierungskommission ersichtlich: Als Vertreter der Deutschen Gesellschaft für Neurochirurgie (DGNC), der Deutschen Gesellschaft für Orthopädie und Orthopädische Chirurgie (DGOOC), der Deutschen Gesellschaft für Nuklearmedizin e. V. (DGNUK), der Deutschen Gesellschaft für Kinderchirurgie e. V. (DGKCH) und der Arbeitsgemeinschaft Pädiatrische Radioonkologie (APRO) sind u. a. Kollegen genannt, die in ihren Bereichen als Referenzexperten fungieren und damit einen intrinsischen Interessenkonflikt bei ihrer Beratung haben könnten.

## Schlussfolgerungen und Ausblick

Wir haben gezeigt, dass die Zertifizierungskriterien, die momentan durch die DKG für die Zertifizierung eines kinderonkologischen Moduls gefordert werden, einer fundierten wissenschaftlichen Grundlage entbehren. Die Auswirkungen ihrer strikten Einhaltung auf die momentan hohe Versorgungsqualität in Deutschland sind unklar, gefährden möglichweise mittel- bis langfristig die medizinische Expertise und erfordern daher, dass überprüfbare Qualitätskriterien, die über die Empfehlung des G‑BA hinausgehen, durch GPOH-initiierte Studien prospektiv geprüft werden.
